# Association between circulating plasma CTRP3 levels and acute ischemic stroke: a systematic review and meta-analysis

**DOI:** 10.3389/fnins.2025.1582743

**Published:** 2025-06-17

**Authors:** Qingsheng Niu, Ziyi Zhu, Yaowen Jiang, Zhi Wan

**Affiliations:** ^1^Department of Emergency Medicine, West China Hospital of Sichuan University, Sichuan University, Chengdu, China; ^2^Laboratory of Emergency Medicine, Disaster Medical Center, West China Hospital of Sichuan University, Chengdu, China; ^3^West China School of Medicine, Sichuan University, Chengdu, China

**Keywords:** CTRP3, ischemic stroke, meta-analysis, adipokine, biomarker

## Abstract

**Background:**

C1q tumor necrosis factor (TNF)-related protein 3 (CTRP3) is a novel adipokine that has been shown to exert neuroprotective effects in acute cerebral infarction. However, conflicting results have emerged regarding the circulating levels of CTRP3 between patients with acute ischemic stroke and healthy individuals. This meta-analysis aims to investigate the association between circulating CTRP3 levels and acute ischemic stroke.

**Objective:**

The objective of this meta-analysis is to re-evaluate the relationship between circulating CTRP3 levels and acute ischemic stroke.

**Methods:**

We conducted a comprehensive search across PubMed, Web of Science, Embase, Cochrane Library, CNKI, VIP, Wanfang Data, and CBM to identify relevant studies up to February 2025 and 110 articles were found. After screening the titles, abstracts, and full texts, a total of 14 articles were ultimately included in this meta-analysis. Due to high heterogeneity, we conducted subgroup analyses stratified by patient characteristics, clinical and biochemical parameters, carotid intima-media thickness, IL-6 levels, CTRP9 levels, smoking, hypertension, and diabetes. Publication bias was evaluated using Egger’s regression test, Begg’s correlation analysis, and funnel plot visualizations.

**Results:**

The results indicated that patients with acute ischemic stroke exhibited significantly lower circulating levels of CTRP3 compared with control group (*Z* = 6.04, *P* < 0.00001). Subgroup analysis revealed that the observed heterogeneity could be attributed to patient age and body mass index (BMI). The year of publication, clinical biochemical parameters, carotid intima-media thickness, IL-6, CTRP9, smoking, hypertension, and diabetes categorization were not sources of heterogeneity.

**Conclusion:**

The meta-analysis confirmed that circulating levels of CTRP3 were significantly lower in patients with acute ischemic stroke compared with control group. This association may be modulated by patient age and BMI.

## Introduction

Acute ischemic stroke (AIS) is a leading cause of disability and death globally, imposing a staggering burden at both individual and societal levels (Katan and Luft, 2018). The complex physiological mechanisms of the brain have led to the current lack of stroke-specific biomarkers (Salaudeen et al., 2024). Thus, it is important to investigate the relevant risk factors following acute ischemic stroke for early detection and prevention in clinical practice.

The novel adipokine complement C1q/tumor necrosis factor-related protein 3 (CTRP3) is associated with inflammation, atherosclerosis, and the regulation of various physiological and pathological processes (Li Y. et al., 2017). CTRP3 is a highly hydrophilic secreted protein, with an N-terminal hydrophobic signal peptide, and no transmembrane domains. CTRP3 was first discovered in 2001 was originally named CORS26 (Collagenous repeat-containing sequence 26 kDa protein) (Maeda et al., 2001). CTRP3 has also been referred to as cartducin and cartonectin, names derived from its observed expression in embryonic cartilage tissue (Maeda et al., 2006). Two splice variants of CTRP3 have been identified to date. The longer isoform, referred to as CTRP3B, results from the retention of intron 1 and includes an additional 73 amino acids at the N-terminus, along with a conserved N-linked glycosylation site not present in the shorter isoform, CTRP3A. Although the specific physiological roles of these variants remain to be elucidated, both isoforms are secreted and detectable in human serum (Peterson et al., 2010). Although CTRP3 has not been definitively confirmed to have a specific receptor, some studies suggest that its functions may be mediated through AdipoR2 (Adiponectin receptor 2) (Murayama et al., 2021). Prior studies have shown AdipoR2 expression in neurons and glial cells in the ischemic penumbra (Clain et al., 2022). In addition, previous studies have demonstrated that CTRP3 can activate the PI3K/Akt, MAPK/ERK, and AMPK signaling pathways, which are typically mediated by membrane receptors, suggesting the existence of an as-yet unidentified binding mechanism between CTRP3 and membrane receptors (Guo et al., 2020).

In recent years, an increasing number of studies have found that CTRP3 is associated with various inflammatory diseases. For example, serum CTRP3 levels are significantly elevated in patients with Hashimoto’s disease compared to controls (Al-Abboody et al., 2025), while circulating CTRP3 levels are markedly reduced in male patients with coronary syndrome (Schmid et al., 2024). Meanwhile, CTRP3 levels are significantly downregulated in asthmatic mice (Lin and Yi, 2023). In addition, in mouse models of rheumatoid arthritis, CTRP3 is markedly increased in two different models (Murayama et al., 2014). In a mouse model of autoimmune encephalomyelitis, CTRP3 inhibits Th17 cell differentiation and exhibits anti-inflammatory effects (Murayama et al., 2021). These evidences enhance the clinical relevance of CTRP3 and support its role as a systemic inflammatory marker. Mounting evidence indicates that inflammatory processes are critically involved in the onset and progression of acute ischemic stroke (Ahmad et al., 2014). Alterations of CTRP3 levels may be associated with the onset of acute ischemic stroke (Sun et al., 2021). Currently, few studies have reported on the changes in serum CTRP3 levels in patients with acute ischemic stroke, with the majority of research focusing on Chinese studies. The predictive value of CTRP3 for acute ischemic stroke remains unclear. In addition, conflicting results have emerged regarding the association between circulating CTRP3 levels and acute ischemic stroke. Therefore, our study aims to explore the relationship between circulating CTRP3 levels and acute ischemic stroke.

## Methods

### Search strategy

Two researchers (Qingsheng Niu and Ziyi Zhu) conducted searches in the following databases: PubMed, Web of Science, Embase, Cochrane Library, CNKI, VIP, Wanfang Data, and CBM. The search period covered the time from the inception of each database through February 2025. No language restrictions were applied during the search process, and reference lists were reviewed to ensure no relevant studies were missed. Our search protocol has been registered on the PROSPERO platform with the registration number: CRD42025640335. The search strategy used was: (CTRP3 OR C1q TNF related protein 3 OR C1q Tumor Necrosis Factor Related Protein 3 OR CORS-26 OR cartducin OR cartonectin) AND (Acute Ischemic Stroke OR Cryptogenic Ischemic Stroke OR Cryptogenic Embolism Stroke OR Cryptogenic Stroke OR Wake-up Stroke). This process was repeated until no further relevant articles were identified. Any discrepancies were resolved through discussion with a third researcher (Yaowen Jiang).

### Criteria

Inclusion criteria: (1) Studies involving adults with or without complications of acute ischemic stroke; (2) Acute ischemic stroke considered as an exposure factor, including patients meeting diagnostic criteria; (3) Control group comprising healthy adults undergoing simultaneous physical examinations during the same period; (4) Circulating levels of CTRP9 evaluated as an outcome measure; (5) Cohort studies and randomized controlled trials.

Exclusion criteria: (1) Articles lacking valid data; (2) Duplicate publications; (3) Retracted articles.

### Data collection and quality assessment

We extracted the following data from the included articles: the number of patients and controls, the first author, year of publication, study region, age, body mass index (BMI), relevant clinical biochemical markers, intimal thickness (IMT), IL-6, circulating CTRP9, the circulating CTRP3 levels, smoking, hypertension, and diabetes in both patients and controls. The risk of bias for all selected studies was assessed using the Newcastle-Ottawa Scale (NOS) (Wells et al., 2000). Each study could score a maximum of 9 points. A score of 0–3 was considered low quality, 4–6 points were classified as moderate quality, and 7–9 points as high quality. Data extraction was performed independently by two researchers, who also assessed the quality of the references, followed by a cross-validation process. A third researcher was involved in discussions to reach a consensus in case of any discrepancies.

### Statistical analysis

We used mean values and standard deviations (SD) to describe the data extracted from the studies, and selected the standardized mean difference (SMD) with a 95% confidence interval (CI) to represent continuous variable data. Heterogeneity was assessed using the Q test and *I*^2^ statistic. An *I*^2^ value of less than 50% indicated no significant statistical heterogeneity, in which case a fixed-effects model was applied. Conversely, if significant heterogeneity was present, a random-effects model was used. Publication bias was evaluated using Egger’s linear regression test, Begg’s rank correlation test, and funnel plots. Furthermore, we conducted a sensitivity analysis by sequentially excluding each study to observe any changes in the direction of the SMD. A *p*-value of less than 0.05 was considered statistically significant in our analysis. Statistical analyses were performed using Review Manager 5.3 and STATA 15.1.

## Results

### Search strategy

**FIGURE 1:**
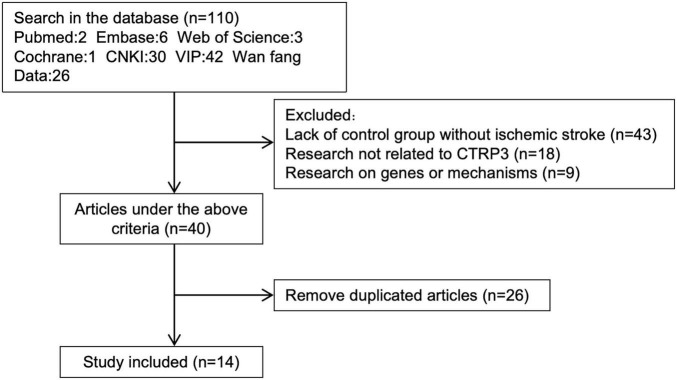
Articles screening process.

A total of 110 articles were initially identified through preliminary screening. After reviewing the titles and abstracts, 40 articles were excluded. Among the 40 excluded articles, 43 lacked a non-AIS control group, 18 were unrelated to AIS, and 9 focused on the genetics or pathogenesis of CTRP3. The remaining 40 articles were thoroughly examined, and those with duplicate data sources or unavailable full texts were further removed. Ultimately, a total of 14 studies, meeting the inclusion and exclusion criteria, were included in the final analysis (Figure 1).

### Study characteristics

This meta-analysis included 14 studies. Among these studies, 13 articles examined the levels of CTRP3 in patients with acute ischemic stroke and compared them with those in a concurrent healthy control group. Additionally, one study assessed the differences in CTRP3 levels between hemorrhagic and non-hemorrhagic transformations following acute cerebral infarction. A detailed summary is provided in Table 1.

**TABLE 1:** Basic characteristics of articles.

Study	*n*		Age		CTRP3 mean		CTRP3 SD	
	Patients	Controls	Patients	Controls	Patients	Controls	Patients	Controls
Liu et al. (2024)	87	85	63.87	62.21	238.34	372.14	65.23	83.47
Yan et al. (2020)	200	200	53	54	87	137	14	36
Li X. et al. (2021)	163	72	63.34	62.84	86.35	140.74	14.51	31.11
Li et al. (2019)	55	55	61.16	62.62	129.65	326.5	53.21	61.06
He et al. (2022)	234	85	64.68	64.23	191.34	247.18	46.25	51.47
Wu et al. (2024)	64	141	63.75	65.61	197.75	260.16	30.45	40.1
Xu et al. (2022)	120	64	65.23	65.33	87.64	113.26	9.17	13.21
Liu et al. (2021)	74	100	70.77	72.18	390.83	313.04	86.43	64.48
Li N. et al. (2021)	56	50	52.64	54.3	127.3	177.8	33.45	54.45
Gao X. et al. (2020)	85	80	69.54	70.12	240.33	393.52	53.58	80.67
Sun et al. (2021)	45	40	–	–	324.56	451.12	74.36	81.65
Zhang et al. (2021)	102	98	64.04	63.87	245.38	380.64	61.06	73.24
Wen et al. (2019)	90	45	55.02	54.37	84.39	134.74	6.71	8.26
Wang et al. (2021)	939	100	59.25	57.32	281.14	385.43	25.95	23.62

*n*, the number of populations in control and patients’ group.

### Quality assessment

The quality of the included studies was assessed using the NOS scale (Table 2). Among the studies, 11 were classified as high quality (78.6%, 11/14), 3 as moderate quality (21.4%, 3/14), and none were rated as low quality.

**TABLE 2:** All primer sequences used in the experiment.

References	Selection of study population	Comparability	Exposure or outcome assessment	Total score
Liu et al. (2024)	4	1	3	8
Yan et al. (2020)	4	1	3	8
Li X. et al. (2021)	2	2	3	7
Li et al. (2019)	2	2	3	7
He et al. (2022)	2	1	2	5
Wu et al. (2024)	3	1	3	7
Xu et al. (2022)	3	2	2	7
Liu et al. (2021)	2	1	2	5
Li N. et al. (2021)	4	1	3	8
Gao X. et al. (2020)	3	2	2	7
Sun et al. (2021)	3	1	3	7
Zhang et al. (2021)	2	2	3	7
Wen et al. (2019)	2	2	2	6
Wang et al. (2021)	4	1	3	8

### Circulating levels of CTRP3

**FIGURE 2:**
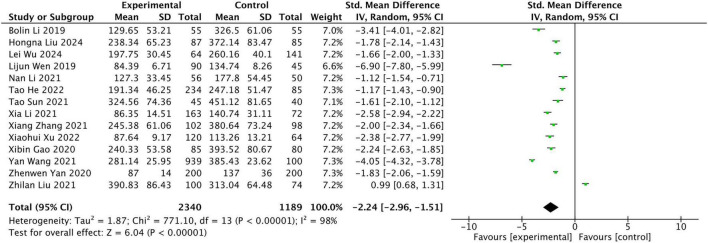
Meta-analysis of Circulating CTRP9 levels in non-AIS and AIS patients.

The results of the meta-analysis indicated that the circulating levels of CTRP3 were significantly lower in AIS patients compared to the control group (*Z* = 6.04, *P* < 0.00001) (Figure 2). Due to the high heterogeneity, statistical analysis was performed using a random-effects model. A Leave-1-out sensitivity analysis was conducted to assess the impact of each individual study on the overall results. Notably, consistent findings were observed both before and after the exclusion of each study.

### Subgroup analysis

Given the high heterogeneity, subgroup analysis was conducted based on the following four factors: (1) key study characteristics (year of publication, patient age, and BMI); (2) relevant clinical biochemical markers [total cholesterol (TC), low-density lipoprotein (LDL), and high-density lipoprotein (HDL)]; (3) intimal thickness; (4) concentrations of CTRP9 and IL-6; (5) smoking, hypertension, and diabetes. Meta-regression was employed to assess the heterogeneity between subgroups (Table 3).

**TABLE 3:** The results of subgroup analysis.

Subgroup	Number of comparisons	Participants	SMD (95% CI)	*Z*-value	*P*	Test of heterogeneity		*P* ^a^
						**I2 (%)**	***P***	
**Overall**	14	3,529	−2.24 [−2.96, −1.51]	6.04	<0.001	98	<0.001	
**1. Year**	14	3,529	−2.24 [−2.96, −1.51]					0.154
Before 2021	4	810	−3.53 [−4.97, −2.08]	4.77	<0.001	98	<0.001	
2021	6	1,839	−1.73 [−3.29, −0.17]	2.17	0.03	99	<0.001	
After 2021	4	880	−1.73 [−2.23, −1.24]	6.89	<0.001	89	<0.001	
**2. Age**	13	3,444	−2.22 [−2.97, −1.46]					0.030
50–60	4	1,680	−3.43 [−5.14, −1.71]	3.92	<0.001	99	<0.001	
60–70	8	1,590	−2.13 [−2.55, −1.70]	9.70	<0.001	91	<0.001	
> 70	1	174	0.99 [0.68, 1.31]	6.12	<0.001	–	<0.001	
**3. BMI**	10	2,189	−2.10 [−2.87, −1.33]					0.045
< 25	8	1,889	−1.55 [−2.30, −0.80]	4.06	<0.001	98	<0.001	
> 25	2	300	−4.55 [−9.11, 0.01]	1.95	0.05	99	<0.001	
**4. TC**	4	724	−3.14 [−4.44, −1.85]					0.514
< 5.2	2	384	−2.18 [−2.54, −1.81]	11.69	<0.001	50	0.16	
> 5.2	2	340	−4.26 [−9.39, 0.87]	1.63	0.1	99	<0.001	
**5. LDL**	4	724	−3.14 [−4.44, −1.85]					0.514
< 3.37	2	340	−4.26 [−9.39, 0.87]	1.63	0.1	99	<0.001	
> 3.37	2	384	−2.18 [−2.54, −1.81]	11.69	<0.001	50	0.16	
**6. HDL**	4	724	−3.14 [−4.44, −1.85]					0.434
< 1.04	2	335	−4.43 [−9.23, 0.37]	1.81	0.07	99	<0.001	
> 1.04	2	389	−2.01 [−2.71, −1.32]	5.66	<0.001	86	0.007	
**7. IMT**	4	907	−3.00 [−4.14, −1.87]					0.447
< 1.36	2	307	−4.32 [−9.33, 0.69]	1.69	0.09	99	<0.001	
> 1.36	2	600	−1.88 [−2.08, −1.69]	19.15	<0.001	0	0.41	
**8. CTRP9**	5	991	−2.72 [−3.75, −1.69]					0.551
< 185.2	2	372	−1.90 [−2.14, −1.65]	15.13	<0.001	0	0.38	
> 185.2	3	619	−3.38 [−5.53, −1.22]	3.08	0.002	99	<0.001	
**9. IL-6**	3	595	−2.26 [−3.15, −1.36]					0.609
< 84.1	2	510	−2.60 [−4.15, −1.04]	3.27	0.001	96	<0.001	
> 84.1	1	85	−1.61 [−2.10, −1.12]	6.41	<0.001			
**10. Smoking**	4	599	−2.32 [−4.69, 0.06]					0.331
High proportion	3	425	−3.42 [−5.77, −1.07]	2.85	0.004	98	<0.001	
Low proportion	1	174	0.99 [0.68, 1.31]	6.12	<0.001			
**11. Hypertension**	3	415	−2.31 [−5.52, 0.91]					0.171
High proportion	2	280	−0.06 [−2.14, 2.02]	0.06	0.95	98	<0.001	
Low proportion	1	135	−6.90 [−7.80, −5.99]	14.88	<0.001			
**12. Diabetes**	3	415	−2.31 [−5.52, 0.91]					0.835
High proportion	2	309	−2.94 [−10.67,4.79]	0.74	0.46	100	<0.001	
Low proportion	1	106	−1.12 [−1.54, −0.71]	5.36	<0.001			

*P*^a^, the results of meta-regression.

The main characteristics of the studies analyzed included the year of publication, patient BMI, and age. Subgroup analysis was performed based on these characteristics. The included studies were published between 2013 and 2024, with 2021 serving as a cutoff. The results indicated no significant heterogeneity between subgroups (*P* = 0.26), suggesting that publication year was not a source of heterogeneity. Regarding patient age, the studies primarily focused on individuals aged 50–80 years. Thus, the studies were divided into three subgroups for analysis: 50–60 years, 60–70 years, and > 70 years. The results revealed statistically significant heterogeneity between the three subgroups (*P* = 0.03), indicating that patient age was a major source of heterogeneity. Subgroup analysis also suggested that CTRP3, being a fat-derived cytokine homologous to adiponectin, might be associated with obesity. According to the international BMI classification, individuals with a BMI < 25 are considered within the normal range, while those with a BMI ≥ 25 are classified as overweight. Our findings indicated that heterogeneity was significant based on BMI (*P* = 0.045).

Subgroup analysis was also performed for relevant clinical biochemical markers, with the results suggesting that TC (*P* = 0.514), LDL (*P* = 0.514), and HDL (*P* = 0.434) were not the primary sources of heterogeneity in this study. Previous research has reported correlations between CTRP3 and the intimal thickness of the internal carotid artery and plasma IL-6 in acute ischemic stroke. Therefore, we conducted subgroup analysis for these parameters, and the results indicated that intimal thickness of the internal carotid artery (*P* = 0.447) and plasma IL-6 (*P* = 0.609) were not major sources of heterogeneity. As CTRP9 is a protein in the same family as CTRP3, and CTRP3 can promote the production of both CTRP9 and adiponectin (Zhang et al., 2017), we performed a subgroup analysis by categorizing CTRP9 into high-concentration and low-concentration groups based on available research data. Our results suggested that CTRP9 (*P* = 0.551) was not a major source of heterogeneity. However, due to the limited number of studies included in the subgroup analysis, further investigation is needed to explore the relationship between CTRP3 and CTRP9 and whether there is any potential synergistic effect between them. We conducted subgroup analyses by categorizing smoking, hypertension, and diabetes into high-prevalence and low-prevalence groups based on the available proportion data. The results of the subgroup analyses suggest that smoking (*P* = 0.331), hypertension (*P* = 0.171), and diabetes (*P* = 0.835) does not appear to contribute to the heterogeneity. However, the sample sizes of these subgroups are relatively small, making the results of the subgroup analyses unstable and susceptible to the influence of individual studies.

### Publication bias

**FIGURE 3:**
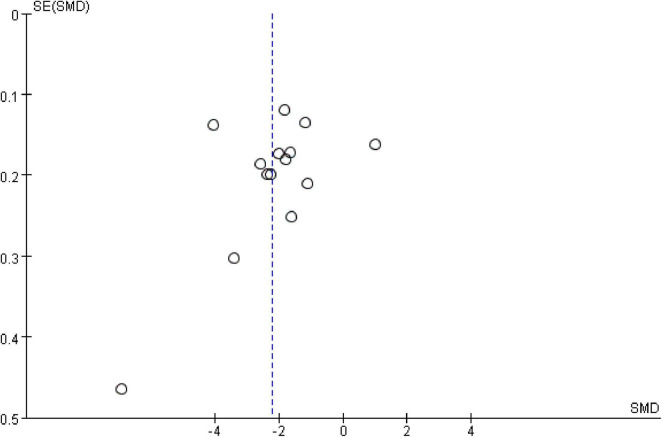
The funnel plot.

The funnel plot depicting the included studies was used to assess publication bias (Figure 3). The results of the Egger test (*t* = −0.96, *P* = 0.356) and Begg test (*z* = −1.48, *P* = 0.139) were not significant. After trim-and-fill adjustment, no studies were imputed, suggesting that the results of the meta-analysis were robust and that there was no evidence of publication bias.

## Discussion

CTRP3 is an important adipokine and a paralog of adiponectin. Due to its unique structure and multi-target characteristics, CTRP3 plays a crucial regulatory role in glucose and lipid metabolism as well as in inflammatory responses. In cardiovascular and cerebrovascular diseases, CTRP3 exerts significant protective effects (Guo et al., 2020). CTRP3 is a potential neuroprotective adipokine. Early studies have detected the presence of CTRP3 in human cerebrospinal fluid, suggesting that CTRP3 is a protein capable of crossing the blood-brain barrier (Schmid et al., 2017). Animal studies have reported that CTRP3 can protect mitochondrial function in hippocampal neurons subjected to hypoxia-reoxygenation through the AMPK signaling pathway, thereby reducing neuronal apoptosis (Gao J. et al., 2020). Moreover, KDM4A can increase MDM2 expression and reduce CTRP3 expression in microglia, thereby promoting polarization and exacerbating brain injury after ischemic stroke in mice. This suggests a neuroprotective role of CTRP3 (Du et al., 2025). Previous studies have suggested contradictory results regarding the plasma concentrations of CTRP3 in patients with acute ischemic stroke and in concurrent healthy control groups. This study analyzed the changes in CTRP3 plasma levels in acute ischemic stroke patients. Our findings indicate that the plasma levels of CTRP3 are significantly lower in patients with acute ischemic stroke compared to the concurrent healthy controls. An animal study suggested that CTRP3 acts as an intermediary signaling molecule, with significant downregulation in brain microvascular endothelial cells induced by oxygen-glucose deprivation/reperfusion (OGD/R). Increased transcription of CTRP3 was found to improve endothelial cell dysfunction induced by OGD/R (Zeng et al., 2023). The above studies confirm that CTRP3 levels are significantly reduced in patients with acute ischemic stroke, suggesting that CTRP3 may have a potential neuroprotective effect on neurons.

Due to the high heterogeneity of this study, we performed a subgroup analysis to explore the factors influencing the different effects of CTRP3. The results indicated that the year of publication was not a source of heterogeneity. However, the age distribution of the population may contribute to the observed heterogeneity. Notably, current research has not yet demonstrated a significant influence of age on CTRP3 concentrations. A multicenter study from China indicated a significant reduction in CTRP3 levels in Alzheimer’s disease-related cognitive impairment, suggesting that CTRP3 may serve as a potential biological marker for neurodegenerative disorders, possibly related to aging (Huang et al., 2024). Future large-scale studies are needed to elucidate the impact of age on plasma CTRP3 concentrations. As an adipokine, CTRP3 levels may be associated with obesity. Research has shown that in obese patients, CTRP3 concentrations are significantly elevated in males, while in females, CTRP3 levels are significantly reduced (Wagner et al., 2016). Our study suggests that normal weight and overweight status are main the sources of significant heterogeneity in CTRP3 levels. Our findings are consistent with previous results, indicating that obesity can influence the expression of CTRP3 in plasma (Guo et al., 2020). Our results support the hypothesis that adipose tissue may be the principal source of circulating CTRP3. Further large-scale studies are needed to confirm the impact of obesity on CTRP3 levels. Additionally, all the populations included in our study were from China, and there are significant genetic, lifestyle, dietary, and regional differences compared to populations from other countries, which may have introduced bias in our conclusions. Clinical research should therefore involve multicenter, multi-regional studies to validate these findings.

CTRP3, like other adipokines, plays a key role in lipid regulation. In the serum of diabetic patients, CTRP3 is positively correlated with HDL and negatively correlated with TC and LDL, highlighting its important regulatory role in lipid metabolism (Li J. et al., 2017). Preclinical experiments have confirmed that short-term administration of recombinant CTRP3 to obese mice fed a high-fat diet significantly modulates lipid metabolism in hepatocytes, leading to a reduction in serum concentrations of TC and LDL in the mice (Peterson et al., 2013). In our study, acute ischemic stroke was stratified based on the different levels of these markers, followed by subgroup analysis. The results indicated that TC, LDL and HDL are not the primary sources of heterogeneity. Although we also extracted data on triglycerides (TG), all the TG values were above the abnormal threshold and closely clustered, so no subgroup analysis was performed for TG. It is noteworthy that, to date, basic stroke research has still lacked investigations into the mechanisms by which CTRP3 regulates lipid metabolism. Future studies are needed to explore the regulatory role of CTRP3 in lipid metabolism.

Intimal thickness of the carotid artery is considered an early indicator of atherosclerosis and may serve as a predictor of cardiovascular disease risk (Simon and Chironi, 2007). Carotid intima-media thickness (IMT) is a sensitive marker for assessing carotid artery atherosclerosis, with thickening occurring early in the process of carotid arteriosclerosis (Sarioglu et al., 2017). The studies included in our analysis consistently indicate a positive correlation between carotid IMT and plasma CTRP3 levels. Consequently, we performed a subgroup analysis on IMT. However, our results suggest that IMT is not a major source of heterogeneity. IL-6 is an upstream inflammatory mediator in atherosclerosis. It activates hepatic cells to produce C-reactive protein, thereby promoting inflammation, enhancing platelet adhesion, and facilitating the development of atherosclerosis (Li et al., 2016). Our study suggests that IL-6 is not a major source of heterogeneity. However, as only three studies were included, further research is needed to explore the relationship between IL-6 and CTRP3. CTRP9 and CTRP3 are both members of the CTRP protein family. Previous reports have shown significant alterations in the concentrations of both CTRP3 and CTRP9 in cardiovascular diseases, although the causal relationships between the two proteins (Gao et al., 2019). There is no consensus on the normal range for CTRP9 in previous reports. In this study, the concentrations were classified into high and low groups based on the mean value. The results suggest that CTRP9 is not a major source of heterogeneity in this study. Therefore, further extensive research is needed to investigate the potential synergistic effects of CTRP9 on CTRP3 and their interactions.

Smoking has been confirmed to promote atherosclerosis, trigger inflammatory responses, and cause vascular endothelial dysfunction (Ishida et al., 2024) and all of which are key mechanisms underlying acute ischemic stroke (de la Riva et al., 2024). CTRP3 itself is closely associated with inflammatory pathways (Li Y. et al., 2017). Our subgroup analysis showed that smoking is not a major source of heterogeneity. Our findings indicate that smoking does not markedly alter circulating CTRP3 concentrations. Hypertension is an independent risk factor for AIS and can lead to chronic vascular remodeling and alterations in the inflammatory microenvironment (Webb and Werring, 2022), which may indirectly regulate CTRP3 levels. Studies have shown that CTRP3 levels are significantly reduced in patients with hypertension, suggesting that CTRP3 may be an independent factor influencing blood pressure and could play an important role in the pathogenesis of both obesity and hypertension (Deng et al., 2015). However, our results suggest that hypertension is not a major source of heterogeneity. Further studies are needed to explore the relationship between hypertension and CTRP3, as well as the underlying mechanisms. Diabetes is also an important risk factor for AIS (Chen et al., 2016), our analysis did not reveal a significant impact of diabetes on the heterogeneity of circulating CTRP3 levels. Given the established role of diabetes in AIS pathogenesis, the absence of a significant effect on CTRP3 levels warrants further investigation. It remains unclear whether this reflects a true lack of association or results from insufficient statistical power.

### Limitations

First, all studies included in this meta-analysis were retrospective, making it difficult to establish a causal relationship between CTRP3 levels and acute ischemic stroke. Thus, prospective cohort studies or randomized controlled trials are needed in the future to validate the observed association. Second, due to the use of different units for CTRP3 concentrations across the included studies, standardized mean differences (SMDs) were employed for analysis. Third, the populations included in this study were all from China, and there are no data from other countries, which may introduce regional bias. Future multicenter and multi-ethnic studies are necessary to validate and generalize the findings beyond the Chinese population. Fourth, with only 14 studies included, it is challenging to draw definitive conclusions. Future research with more precise and comprehensive data may provide more robust findings. We have also stated our plan to continue monitoring the literature and conduct an updated analysis once more data become available.

## Conclusion

In conclusion, our meta-analysis demonstrates that circulating CTRP3 levels are significantly lower in patients with acute ischemic stroke compared to healthy controls. This association may be influenced by patient age and BMI. Our findings suggest a potential neuroprotective role for CTRP3 in acute ischemic stroke, aligning with previous preclinical research. However, due to the observational nature of the included studies and their focus on Chinese populations, further large-scale, multicenter, and multinational studies are warranted to confirm the role of CTRP3 in ischemic stroke and its potential as a therapeutic target.
